# Transcriptome profiling revealed multiple circadian rhythm-related genes associated with common gynecological cancers

**DOI:** 10.3389/fonc.2025.1422122

**Published:** 2025-01-29

**Authors:** Lan Peng, Meiping Jiang, Kangming Li, Shuhui Yu, Chunfang Zhao, Lan Zhang, Lan Li

**Affiliations:** Department of Radiation Oncology, The Third Affiliated Hospital of Kunming Medical University (Yunnan Cancer Hospital, Yunnan Cancer Center), Kunming, China

**Keywords:** gynecological cancers, circadian rhythms, prognosis, biomarkers, function, regulatory mechanism

## Abstract

**Background:**

Studies have shown that more than half of the human genome expression is affected by circadian rhythms, which includes genes involved in cell cycle control, DNA repair and apoptosis that are critical in cancer biology. However, the roles of circadian rhythm-related genes (CRRGs) in cervical cancer (CC) and other common gynecologic cancers remain unclear.

**Methods:**

The transcriptome data and clinical information related to CC and other common gynecologic cancers were extracted from the UCSC Xena and Gene Expression Omnibus (GEO) databases. In this study, the differentially expressed CRRGs of CC (target genes) were obtained, and the functional enrichment analysis of these target genes was performed by “clusterProfiler”. Then, the biomarkers of CC were screened out to construct the survival risk model (risk score). Moreover, function and tumor micro-environment (TME) analyses in different risk groups were performed for further study of the potential mechanism of CC. Furthermore, the prognostic value and function analyses of biomarkers in three common gynecologic cancers were performed to reveal the potential agreement or heterogeneity regulations.

**Results:**

A total of 19 target genes were associated with pyrimidine metabolism. The survival risk model was constructed with six biomarkers, including APOBEC3B, CDA, HELLS, RHOB, SLC15A3, and UPP1. Among these, APOBEC3B, HELLS, and SLC15A3 were identified as positive factors, while CDA, RHOB, and UPP1 were identified as negative factors in CC. It is notable that multiple immune-related signaling pathways were associated with the clinical risk of CC, and the immunotherapy sensitivity was worse in the high-risk group. In addition, we found that most of biomarkers had the prognostic values in other common gynecologic cancers. It was notable that the mechanisms by which these biomarkers influence gynecologic cancers were associated with extracellular matrix (ECM) receptor interaction, focal adhesion, etc.

**Conclusion:**

This study identified six circadian rhythm-related biomarkers, including APOBEC3B, CDA, HELLS, RHOB, SLC15A3, and UPP1, which were associated with the prognosis of CC. The mechanisms by which these biomarkers influence gynecologic cancers were associated with ECM receptor interaction, focal adhesion, and other functions. These findings might help to deepen the understanding of the agreement or heterogeneity of CRRGs in the pathological processes of common gynecologic cancers.

## 1 Introduction

Cervical cancer (CC), endometrial cancer, and ovarian cancer are three common gynecologic cancers that pose a significant threat to the health of women worldwide, resulting in substantial economic burdens (1). CC is ranked as the fourth most frequently diagnosed cancer and the fourth leading cause of cancer death in women, with 604,127 new cases of cervical cancer and 341,831 cancer deaths worldwide in 2020. Endometrial cancer and ovarian cancer are the sixth and eighth most common cancers in women globally, respectively, which is also in the forefront of the incidence in the global female population (2). The accelerated process of population aging has resulted in an overall increase in the incidence rate of gynecologic cancers in China in recent years (3, 4). Despite notable advancements in conventional therapeutic modalities, including surgery, radiotherapy, and chemotherapy, the dismal prognosis of recurrent and advanced gynecologic cancer patients persists as a significant challenge in clinical practice (5, 6). Targeted therapy, biological therapy, and immunotherapy have made breakthrough advances in the treatment of this portion of refractory cervical cancer (7, 8). Therefore, it is necessary to identify accurate prognostic biomarkers and molecular targets to achieve accurate and personalized treatment of gynecologic cancer patients.

The term “circadian rhythms” is used to describe the periodic changes in biochemical, physiological, and behavioral functions that occur in almost all eukaryotic organisms in order to adapt to the 24-hour rotation period of the Earth (9). Recent studies have demonstrated that alterations in the expression of circadian rhythm-related genes (CRRGs) are associated with an increased risk, progression and poor prognosis of various diseases, including cancer (10–12). The core circadian rhythm genes Per2 and Bmal1 have been demonstrated to act in a synergistic manner to promote lung tumorigenesis in conjunction with Kras and p53. A deficiency in these genes results in elevated expression of the oncogenic transcription factor c-Myc, which in turn drives glycolysis and glutaminolysis, thereby promoting the proliferation of tumor cells (13). The rhythmic gene NFIL3 has been demonstrated to promote the proliferation and metastasis of tumor cells through the inhibition of NFKBIA transcription and the subsequent enhancement of NF-κB signaling activity (14). A number of circadian rhythm genes have been linked to the prognosis of various cancers, including head and neck squamous cell carcinoma, breast cancer, and liver cancer, have been identified (15–18). In recent years, it has been found that the expression of multiple CRRGs, with PER2 being the most notable, is reduced in CC cells (19). However, there is currently no research examining the prognostic value of CRRGs in CC and other common gynecological cancers.

In this study, we pioneered an innovative approach integrating comprehensive bioinformatics analysis and public data to identify a set of CRRGs that exhibit unique expression patterns in these cancers compared to healthy tissues. Furthermore, a new prognostic model of CC has been constructed, and the relationship between CRRGs and immunotherapy and the tumour microenvironment has been deeply explored. The objective was to gain further insight into the functional mechanisms of CRRGs and to investigate their functional roles in other common gynecologic cancers. This was done in order to address the persistent gaps in understanding the complex interactions between CRRGs and gynecologic cancers, with the aim of developing a deeper understanding of the disease and of therapeutic strategies.

## 2 Materials and methods

### 2.1 Data extraction and pre-processing

In this study, 1,280 CRRGs were obtained from the Circadian Gene Database (CGDB) (**Supplementary Table 1**). The RNA sequencing data, mutation data, survival and clinical information of CC, ovarian serous cystadenocarcinoma (OV) and uterine corpus endometrial carcinoma (UCEC) were downloaded from the Gene Expression Omnibus (GEO), Figshare and UCSC Xena databases. Among them, the GSE7803 dataset contains 21 CC and 10 healthy control (HC) cervical epithelium samples, and the GSE9750 dataset contains 33 CC and 24 HC cervical epithelium samples. These two datasets were combined and removed batch effect by “sva” R package (version 3.44.0), and the combined dataset was utilized for screening the differentially expressed genes (DEGs). The TCGA-CC dataset contains 291 CC samples with survival information, which was used as the training dataset to screen the biomarkers and build the survival risk model. The CGCI-HTMCP-CC dataset contains 117 CC samples with survival information, which was used as the validation dataset to verify the availability of survival risk model. Furthermore, the GSE168652 dataset contains 1 CC and 1 HC tissue samples, which was the single-cell sequencing dataset and was used to study the cellular localization of the biomarkers. Besides, the TCGA-OV dataset contains 373 OV samples, and the TCGA-UCEC dataset contains 543 UCEC samples with survival information, which were further used to evaluate the prognostic value and function of biomarkers in other common gynecologic cancers.

### 2.2 Functional enrichment analysis of target genes

In this study, we employed the R package “limma” to identify differentially expressed genes (DEGs) with thresholds set at (|log_2_FC| > 1.5, adj.*p*.value < 0.05). We subsequently compared these DEGs between the 54 cancerous cell (CC) and 34 healthy cell (HC) samples within the merged dataset (20). Then, the differentially expressed CRRGs (target genes) were obtained by intersecting the DEGs and CRRGs using “venn”. Besides, the functional enrichment analysis of these target genes was conducted by “clusterProfiler” R package (version 4.2.2) (adj.*p*.value < 0.05) (21).

### 2.3 Screening of the biomarkers and construction of the survival risk model of CC

In this study, the biomarkers of CC that obtained by univariate cox and least absolute shrinkage and selection operator (LASSO) analyses were screened for constructing the survival risk model (risk score). We utilized univariate Cox regression, also known as univariate Cox proportional hazards modeling, which was a widely adopted statistical technique for examining the impact of a single independent variable on the hazard of an event within survival data. We assessed the validity of the proportional hazards assumption through the application of the Schoenfeld residual test. Additionally, we implemented LASSO regression, known as Least Absolute Shrinkage and Selection Operator, a prevalent regression method designed to manage high-dimensional datasets. By incorporating a regularization term into the regression analysis, LASSO enabled us to both select relevant variables and mitigate issues related to coefficient shrinkage and multicollinearity. The risk score was calculated by the algorithm: Riskscore = β_1_X_1_ + β_2_X_2_ +… + β_n_X_n_. The Kaplan-Meier (K-M) survival curve, risk curve and receiver operating characteristic (ROC) curve were used to predict the accuracy of survival risk model. And the survival of different groups was compared by Log-Rank test. Moreover, the validation dataset (CGCI-HTMCP-CC dataset) was used to verify the applicability of this survival risk model. Besides, the correlations between risk score and different clinical characteristics (age, stage, grade, pathologic T, M and N) were compared by “wilcoxon”.

### 2.4 Function and tumor micro-environment analyses

In this research, we employed GSVA (Gene Set Variation Analysis), an R package designed to assess the enrichment of gene sets across various samples. The toolkit encompassed a range of methodologies, among which ssGSEA (Single-sample Gene Set Enrichment Analysis) stood out as a non-parametric and unsupervised approach. This method allowed us to compute enrichment scores for gene sets independently within each individual sample. By utilizing these analytical tools, we were able to gain insights into the differential expression patterns of gene sets and their potential implications for biological processes and disease states. The gene-set variation analysis (GSVA) was utilized for studying the Gene Ontology (GO) functions and Kyoto Encyclopedia of Genes and Genomes (KEGG) pathways of the genes in different risk groups by “GSVA” R package (version 1.42.0) (22). On the other hand, the proportions of 28 immune cells and 17 immune reactions between different risk groups were calculated by “single sample gene set enrichment analysis (ssGSEA)” algorithm and compared by “wilcoxon”, respectively. The Wilcoxon test is a nonparametric statistical test used to compare the difference in medians between two independent groups of samples. It is applied to the assumption that the data do not satisfy a normal distribution. Next, the correlations between biomarkers and differential immune cells/reactions, and the correlations between risk score and differential immune cells/reactions were calculated by “spearman”, respectively. Then, the differences of 48 immune checkpoints between different risk groups were compared for assessing the immune reaction. Moreover, the immunophenotype (IPS) score and tumor immune dysfunction and exclusion (TIDE) score were calculated for assessing the immunotherapy sensitivity. Furthermore, the stromal score and immune score were calculated for assessing the tumor purity.

### 2.5 Patient cohorts and immunohistochemistry

The study was approved by the Ethics Committee of Yunnan Cancer Hospital (KYCS2024-276). We enrolled 20 recurrent or metastatic cervical cancer patients who were treated with immune checkpoint inhibitors including Cadonilimab, Zimberelimab and Toripalimab plus chemotherapy or chemoradiotherapy from January 2022 to April 2024. All patients had had formalin-fixed and paraffin-embedded (FFPE) tissues for immunohistochemistry and imaging data for monitoring tumor response to immunotherapy. 16 patients were classified as have clinical complete response (cCR) and 4 patients were classified as progression disease (PD). Patients with partial respond (PR) and stable disease (SD) were not included.

After antigen retrieval, 4 um thick FFPE sections were incubated with primary antibodies against SLC15A3 (1:100, PA5-48477, ThermoFish, America) or CD8 (ready-to-use reagent, ZA-0508, ZSGB-BIO, China) at 4℃ overnight. The next day, slides were incubated with horseradish peroxidase conjugated secondary antibody for 30 min at 37℃, followed by detecting using 3,3′-diaminobenzidine (DAB). SLC15A3 staining was measured using the H-score method. The H-score were calculated as follows: (3 × percentage of strongly stained tumor cells) + (2 × percentage of moderately stained tumor cells) + (percentage of weakly stained tumor cells), ranging from 0 to 300. The density of CD8^+^ lymphocytes in the tumor were measured by quantifying positively stained cells in five random square areas (1 mm^2^ each).

### 2.6 Drug sensitivity analysis

In addition, the maximum inhibitory concentration (IC50) of 138 chemotherapy drugs in different risk groups was compared to study the drug sensitivity of CC by “pRRophetic” R package (version 0.5) (23).

### 2.7 Cellular localization analysis of biomarkers

In this study, quality control of the single-cell dataset (GSE168652) was studied by the “Seurat” R package (version 4.1.0) (genes in cell > 200, cell coverage number > 3, 200 < nFeature_RNA < 5,000, nCount_RNA < 12,000, and percent.mt < 15%) (24). The top 2,000 highly variable genes were screened using “FindVariableFeatures” function. The principal component were clustered by “UMAP”, and the major cell types were annotated by “SingleR” R package (version 1.8.0) (25). Based on these analyses, the expressions of biomarkers in major cell types were calculated for explaining the cellular localization.

### 2.8 Prognostic value analyses of the biomarkers and clinical correlation analyses in three common gynecologic cancers

On the one hand, the K-M survival curves of all biomarkers in OV and UCEC were plotted to determine the prognostic value of biomarkers in common gynecologic cancers by “survminer” R package (version 0.4.9). On the other hand, the expressions of all biomarkers in different clinical characteristics groups of CC, OV, and UCEC were compared to study the clinical correlation, respectively. Among them, the clinical characteristics of CC contains age, stage, grade, pathologic T, M and N, and the clinical characteristics of OV and UCEC contain age, stage, grade.

### 2.9 Function enrichment and mutation analyses of biomarkers in three common gynecologic cancers

The median expression value of each biomarker was used to divide the samples into high and low expression groups, and gene set enrichment analysis (GSEA) was performed to study the KEGG pathways of each biomarker by “clusterProfiler” R package in TCGA-CC dataset, TCGA-OV dataset, and TCGA-UCEC dataset, respectively (|NES| > 1, NOM P < 0.05, and q < 0.25) (20). Besides, the mutation situation of all these biomarkers in the three cancers were analyzed by “TCGAmutations” R package (version 0.3.0), respectively.

### 2.10 Statistical analysis

All analyses were conducted using R language. Differences between two groups were compared by “Wilcoxon” test. If not specified above, *p* < 0.05 was regarded as statistically significant.

## 3 Results

### 3.1 19 Target genes were associated with pyrimidine metabolism

**Figure 1:**
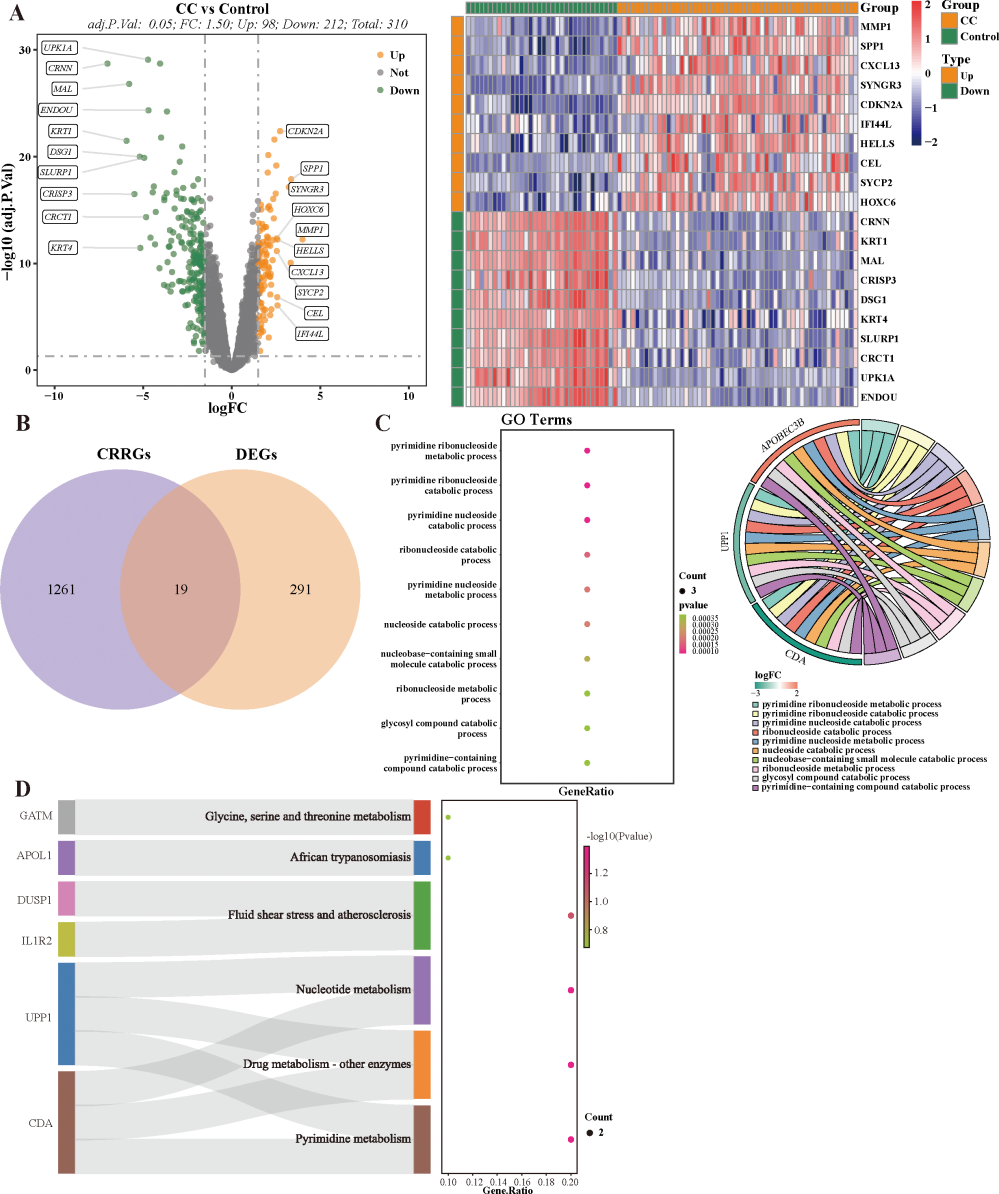
Identification and Functional enrichment analysis of candidate CRRGs. **(A)** Volcano plot and heat map of differentially expressed 310 DEGs. **(B)** Venn diagram of CRRGs and DEGs between cervical cancer and healthy control. **(C, D)** Results of GO and KEGG nrichment pathways.

There were 310 DEGs (98 up-regulated and 212 down-regulated) between 54 CC and 34 HC samples in combined dataset (**Figure 1A**). Then, totals of 19 target genes, including APOBEC3B, APOL1, CDA, DUSP1, EMP1, ESR1, FCGBP, GATM, KANK1, HELLS, IL1R2, MAL, RHCG, RHOB, SLC15A3, SNX10, UPP1, ZNF135, ZSCAN18 were obtained by intersecting 310 DEGs and 1,280 CRRGs (**Figure 1B**). As the perspective of function, these 19 target genes were significantly enriched to pyrimidine ribonucleoside catabolic process, glycosyl compound catabolic process, and etc. 279 GO functions. Besides, these target genes were associated with pyrimidine metabolism, glycine, serine and threonine metabolism, and etc. six KEGG pathways (**Figures 1C, D**, **Supplementary Tables 2, 3**).

### 3.2 Six biomarkers were used to construct the survival risk model of CC

**Figure 2:**
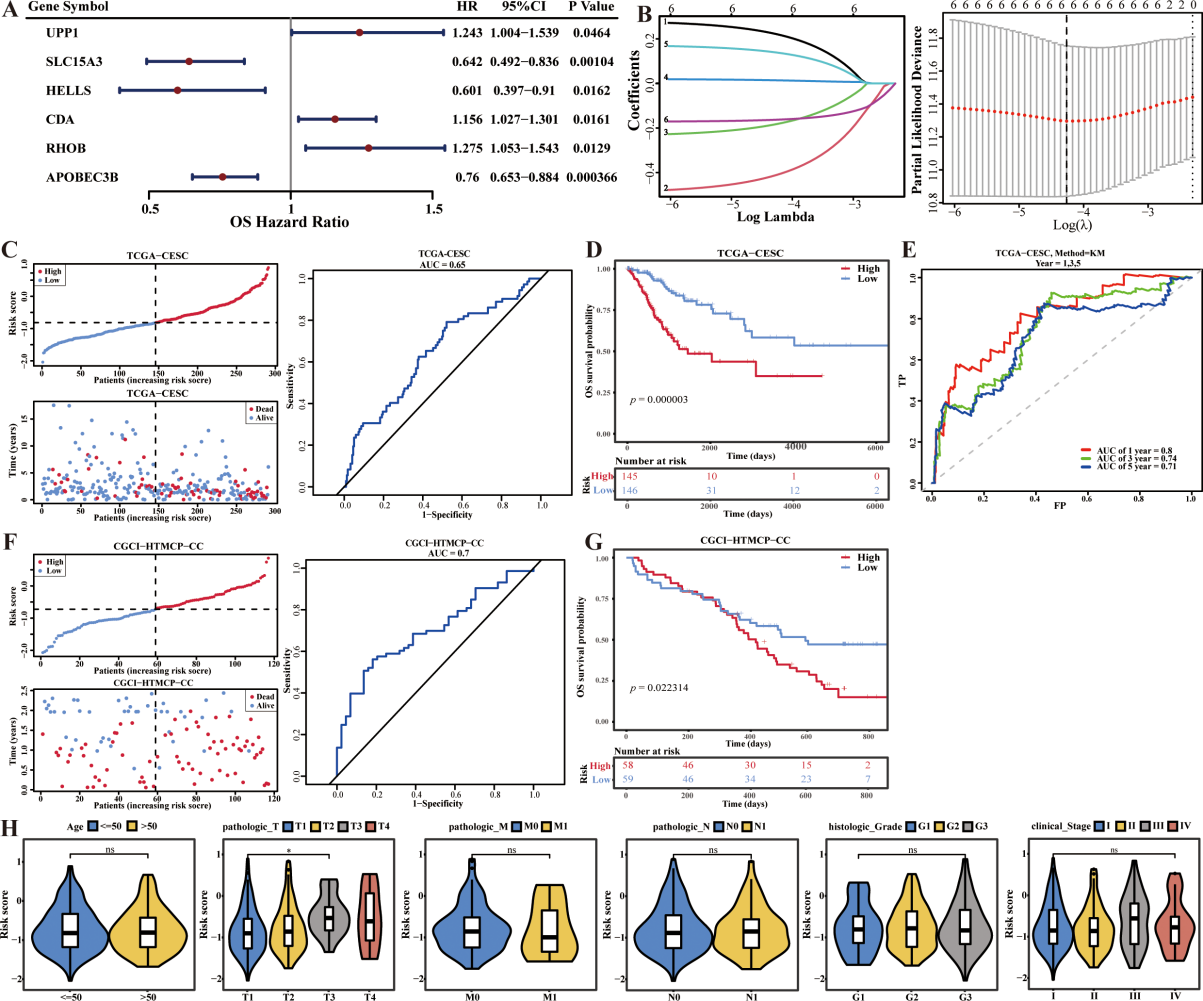
Survival risk model of CC construction, validation and evaluation. **(A)** Forest plot for univariate Cox regression analysis. **(B)** Cross-validation curve and lasso coefficient curves of LASSO regression. **(C)** Risk curve, survival status and ROC curve analysis of risk model in the training set. **(D)** K-M curve compares the survival between two risk groups in the training set. **(E)** Time-dependent ROC curves analysis in the training set. **(F)** Risk curve, survival status and ROC curve analysis of risk model in the validation set. **(G)** K-M curve compares the survival between two risk groups in the validation set. **(H)** Correlations analysis of risk scores and clinical traits.

In this study, six biomarkers, including APOBEC3B, CDA, HELLS, RHOB, SLC15A3, and UPP1 were identified, among them, APOBEC3B, HELLS, and SLC15A3 were positive factors (Hazard Ratio < 1), and CDA, RHOB, and UPP1 were negative factors (Hazard Ratio > 1) of CC (**Figures 2A, B**).Then, the survival risk model was constructed by the algorithm: risk score = -0.1629 × APOBEC3B + 0.0142 × CDA - 0.1827 × HELLS + 0.1390 × RHOB - 0.4112 × SLC15A3 + 0.2217 × UPP1. The risk curve and K-M curve showed that there were significant survival differences between these two risk groups (*p* = 0.000003) (**Figures 2C, D**). Besides, the area under ROC curves (AUC values) was higher than 0.7 (**Figure 2E**). Moreover, the CGCI-HTMCP-CC dataset was used to verify the applicability of this survival risk model. The results of risk curve and K-M curve were consistent with the training dataset (**Figures 2F, G**). These results indicated that this survival risk model was applicable for CC. Besides, the risk score was significantly different between pathologic T1 and pathologic T3 (*p* < 0.05) (**Figure 2H**).

### 3.3 Multiple immune-related signaling pathways were associated with clinical risk of CC

**Figure 3:**
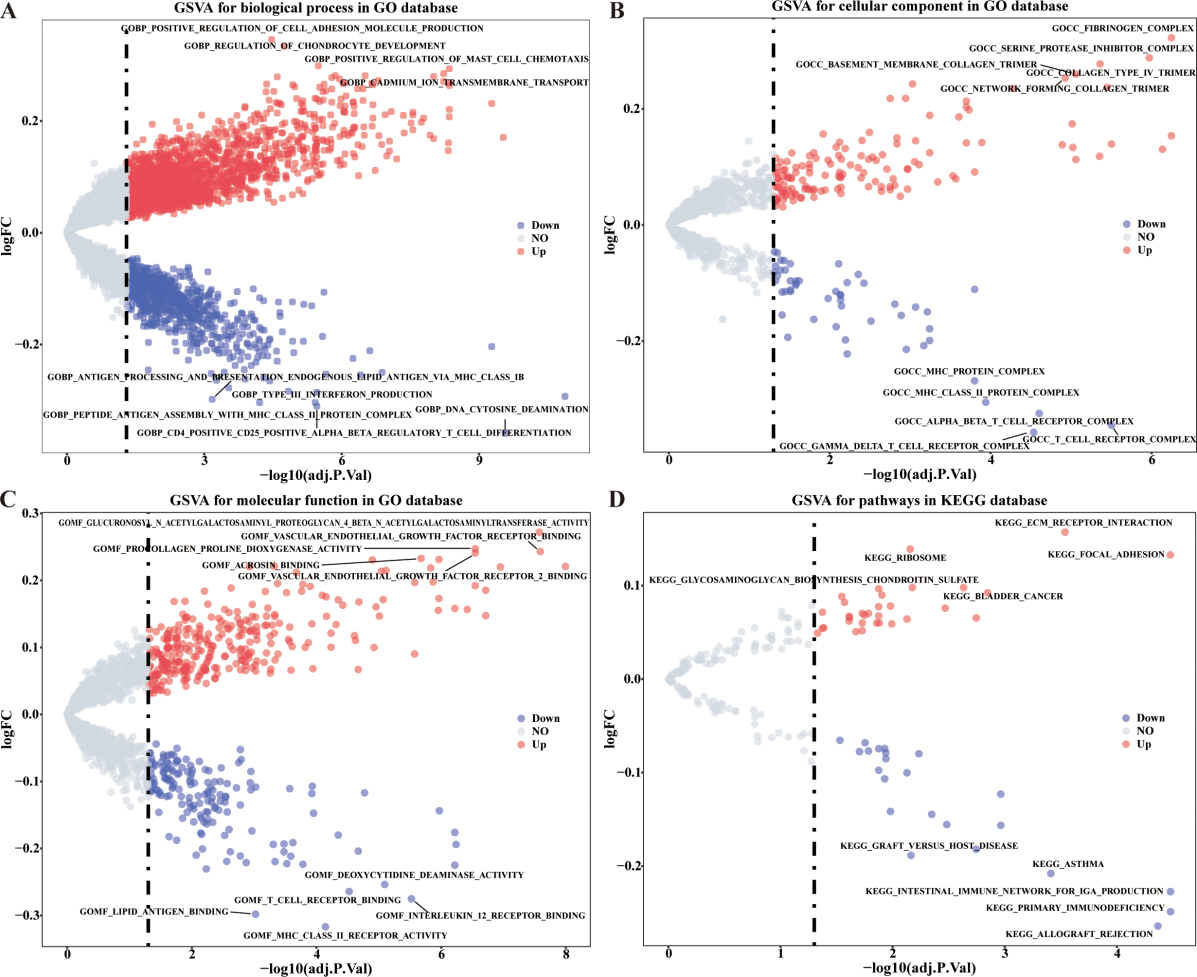
GSVA analysis of differentially expressed CRRGs in different risk groups. **(A–C)** Enriched items in GO analysis. **(D)** Enriched items in KEGG pathway analysis.

As the perspective of GO functions, the positive regulation of mast cell chemotaxis, positive regulation of cell adhesion molecule production, interleukin 21 (IL-21) production, BMP signaling pathway, and etc. functions were significantly up-regulated, and the negative regulation of interferon alpha (INF-α) production, microglial cell migration, IL-27 mediated signaling pathway, and etc. functions were significantly down-regulated in high risk group (**Figures 3A–C**, **Supplementary Table 4**). As the perspective of (KEGG) pathways, the extracellular matrix (ECM) receptor interaction, focal adhesion, TGF beta signaling pathway and etc. 46 pathways were up-regulated, and B cell receptor signaling pathway, T cell receptor signaling pathway, and etc. 30 pathways were down-regulated in high risk group (**Figure 3D**, **Supplementary Table 5**).

### 3.4 The level of immune escape was higher and the immunotherapy sensitivity was worse in high-risk group

**Figure 4:**
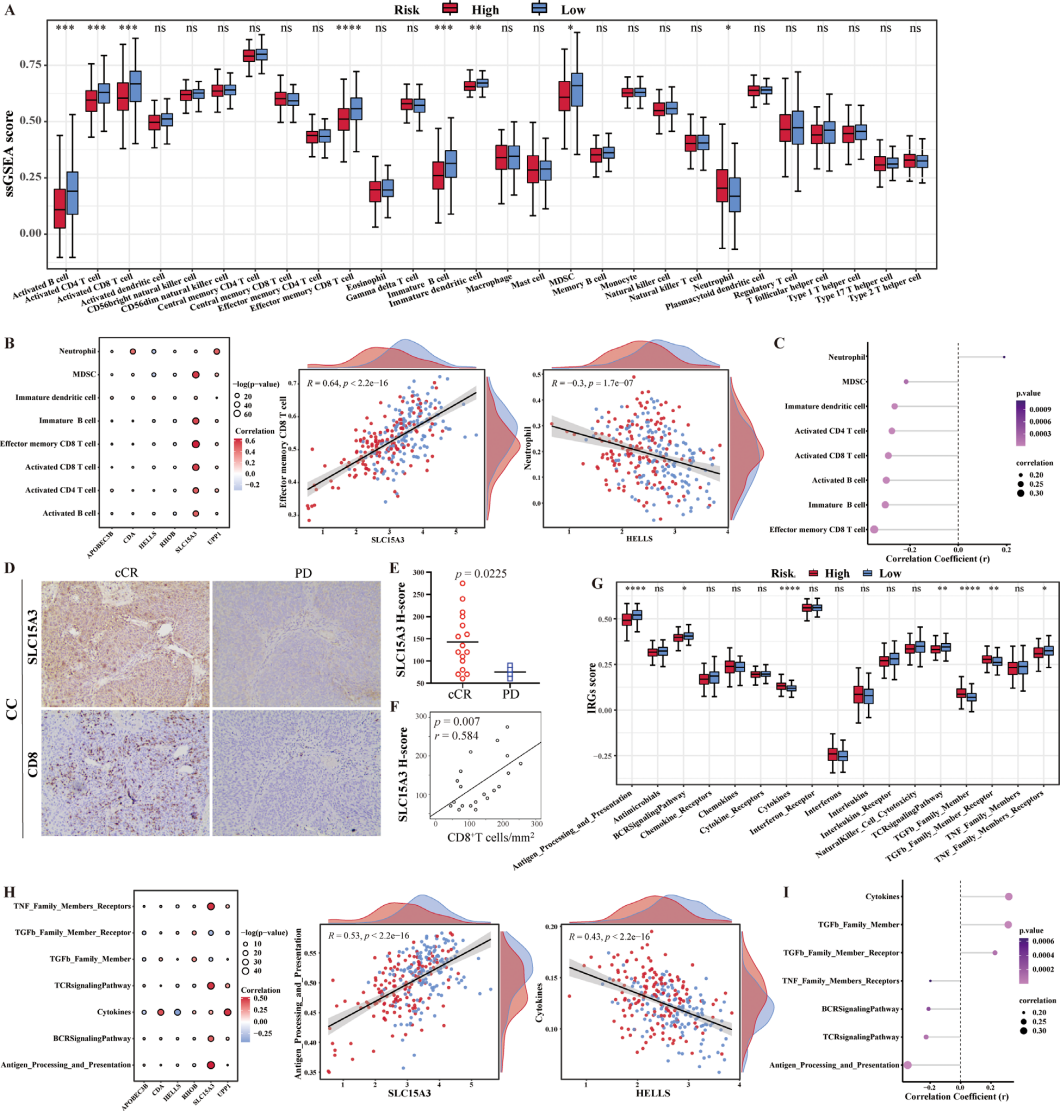
Characteristics of tumor immune cell infiltration and immune reactions in different risk groups. **(A)** The box plot showed the difference in immune cells infiltration between the two risk groups. **(B)** Spearman correlation analysis showed the relationship between differential immune cells and 6-CRRGs. **(C)** Analysis of the correlation between risk score and differential immune cells. **(D)** Representative immunohistochemical images of SLC15A3 and CD8 from tumors of progression disease (PD) and clinical complete response (cCR) cervical cancer patients. **(E)** SLC15A3 H-scores in cCR and PD cervical cancer patients. **(F)** Spearman correlation between CD8+ cell densities and SLC15A3 H-scores in cervical cancer patients. **(G)** The box plot showed the difference in immune reactions between the two risk groups. **(H)** Spearman correlation analysis showed the relationship between immune reactions and 6-CRRGs. **(I)** Analysis of the correlation between risk score and differential immune reactions. *p < 0.05, **p < 0.01, ***p < 0.001, ****p <0.0001, ns: No statistical significance.

In this study, 28 immune infiltrating cell enrichment scores for CESC samples in the training set were calculated based on the ssGSEA algorithm using the R package “GSVA”. The 28 immune infiltrating cell enrichment scores were plotted as box plots based on comparisons between high and low risk samples. There were seven significantly decreased immune cells (activated B cell, activated CD4 T cell, activated CD8 T cell, effector memory CD8 T cell, immature dendritic cell, immature B cell, and MDSC) and only one increased immune cell (neutrophil) in high risk group (*p* < 0.05) (**Figure 4A**). Among them, SLC15A3 was significantly positively correlated with the majority of differential immune cells, which perhaps implied that the key mechanism of SLC15A3 in CC was associated with immune-related functions. In addition, there was the strongest significantly positive correlation between SLC15A3 and effector memory CD8 T cell (R = 0.64, *p* < 2.2e-16), and the strongest significantly negative correlation between HELLS and neutrophil (R = -0.3, *p* = 1.7e-07) (**Figure 4B**). Spearman correlation analysis was performed to analyze the correlation between 8 differential immune infiltrating cells and risk scores. The results showed that the risk score was significantly positively correlated with the proportions of neutrophil, and was significantly negatively correlated with other seven immune cells decreased in high risk group, which was consistent with their expression results (**Figure 4C**).

Considering the strong positive correlation between SLC15A3 and CD8+ T cell, we hypothesized that CC patients with high SLC15A3 expression may benefit from immunotherapy. Immunohistochemistry was used to delineate the SCL15A3 protein expression and CD8+ T cell immune infiltrates in a CC immunotherapy cohort in our department. We found higher H-scores of SLC15A3 in the clinical complete response (cCR) CC patients compared with the progression disease (PD) CC patents (p = 0.0225) (**Figures 4D, E**). The Spearman correlation again demonstrated a positive correlation between SLC15A3 expression and CD8+ T cell densities in CC patients (p = 0.007, r = 0.584) (**Figure 4F**). Future investigations should be tested to explore whether SLC15A3 could serve as a predictive biomarker for immunotherapy in CC patients.

In addition, 17 immune response pathway enrichment scores were plotted as box plots based on comparisons between high and low risk samples. The expression of three immune responses (cytokines, TGFb family members and TGFb family member receptors) was found to be significantly higher in the high-risk group than in the low-risk group, whereas antigen processing and expression, the BCR signaling pathway, the TCR signaling pathway, and the TNF family member receptor were significantly higher in the low-risk group than in the high-risk group (p<0.05) (**Figure 4G**). Seven differential immune response pathways and six risk model genes were subjected to Spearman correlation analysis. Among them, Antigen_Processing_and_Presentation had the strongest positive correlation with SLC15A3, with a correlation coefficient of 0.53. Cytokines had the strongest negative correlation with HELLS, with a correlation coefficient of -0.43 (**Figure 4H**). Similarly, the correlation results between risk score and differential immune reactions were consistent with their expression results (**Figure 4I**).

**Figure 5:**
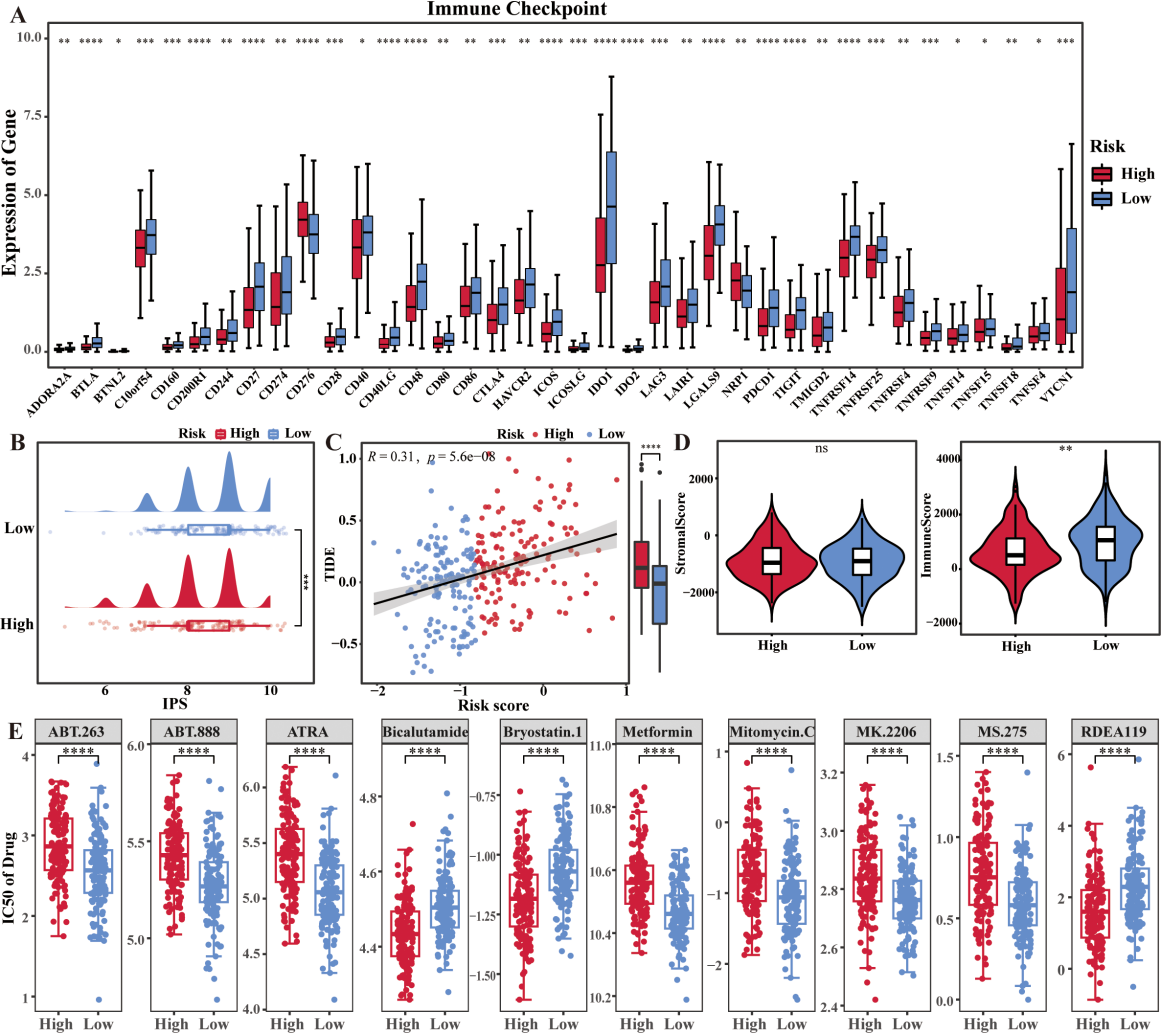
Immune escape levels and immunotherapy sensitivity in two risk groups of cervical cancer patients. **(A)** Box plots showed the differences of immune checkpoints in the two risk groups. **(B–D)** IPS score, TIDE score, stromal score and immune score in the two risk groups. **(E)** Drug sensitivity analysis in the two risk groups. *p < 0.05, **p < 0.01, ***p < 0.001, ****p <0.0001, ns: No statistical significance.

In this study, 48 immune checkpoint molecules extracted from the literature. Compare the differences in expression of the 48 immune checkpoint molecules in the training set between high and low risk groups. Thirty-eight immune checkpoints were found to be significantly different between risk groups, with most of the immune checkpoints in the high-risk group being lower than those in the low-risk group (**Figure 5A**). Notably, the IPS score was significantly lower and the TIDE score was significantly higher in high risk group (*p* < 0.05) (**Figures 5B, C**). In addition, the immunization scores were significantly higher in the low-risk group than in the high-risk (*p* < 0.05) (**Figure 5D**). All these results revealed that the level of immune escape was higher in high risk group and the immunotherapy sensitivity/response was better in low risk group.

In addition, the samples in high risk group were more sensitive to *Bryostatin.1*, *Bicalutamide*, and etc., and the samples in low risk group were more sensitive to *Metformin*, *Mitomycin.C* and etc. (*p* < 0.001) (**Figure 5E**).

### 3.5 Cellular localization analysis of biomarkers

**Figure 6:**
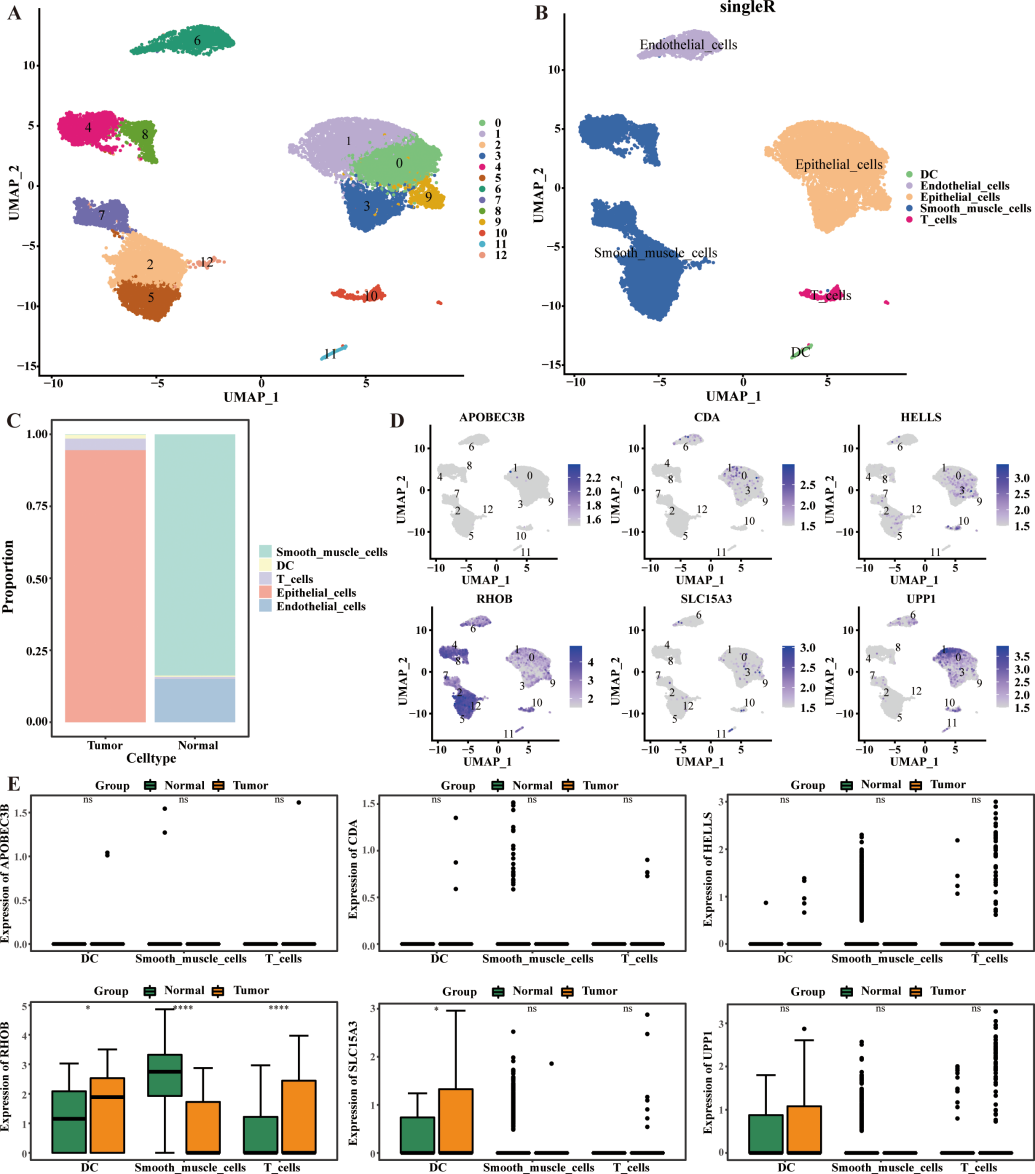
Cellular localization analysis of 6-CRRGs. **(A, B)** UMAP visualization in all the cells displayed with different colors for clusters and cell lineages. **(C)** The proportions of various cells in CC and normal cervical tissue. **(D, E)** Expression of 6-CRRGs in major cell types.

In this study, cells were classified into 13 clusters and identified as five major cell types, including dendritic cell (DC), endothelial cells, epithelial cells, smooth muscle cells, and T cells (**Figures 6A, B**). Among them, the proportion of epithelial cells was highest in CC sample, and the proportion of smooth muscle cells was highest in HC sample (**Figure 6C**). Notably, UPP1 was predominantly localized in epithelial cells, while RHOB was predominantly localized in smooth muscle cells. Compared with healthy controls (HC), RHOB was significantly less expressed in smooth muscle cells, but significantly more expressed in dendritic cells (DC) and T cells in ovarian cancer (OV). Similarly, SLC15A3 was significantly more expressed in DC in OV (*p* < 0.05) (**Figures 6D, E**).

### 3.6 The biomarkers also have the prognostic values in other common gynecologic cancers

**Figure 7:**
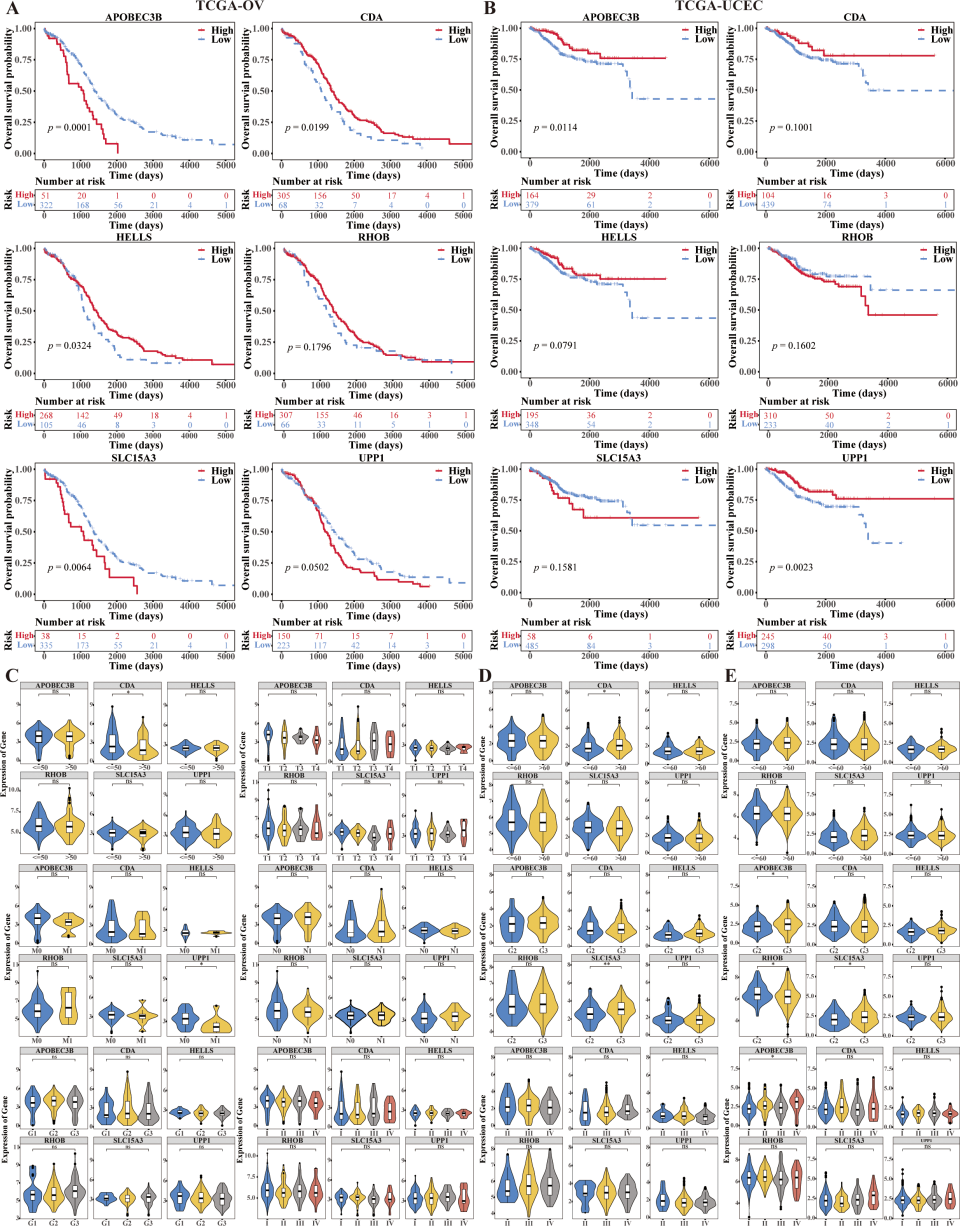
Prognostic value of 6-CRRGs in ovarian cancer patients and uterine corpus endometrial carcinoma patients. **(A)** Survival analysis of 6-CRRGs in OV patients between two risk groups. **(B)** Survival analysis of 6-CRRGs in UCEC patients between two risk groups. **(C–E)** Correlation between the expression of 6-CRRGs and clinical characteristics in CC, OV, UCEC patients.

We used the R package “survminer” to plot the KM curves of model genes based on high and low expression, and to investigate the correlation between model genes and prognosis. The K-M survival analyses results showed that CDA and HELLS were the positive factors (*p* = 0.0199, *p* = 0.0324), and APOBEC3B and SLC15A3 were also the negative factors (*p* = 0.0001, *p* = 0.0064) of OV (**Figure 7A**), APOBEC3B and UPP1 were the positive factors (*p* = 0.0114, *p* = 0.0023) of UCEC (**Figure 7B**). Model gene expression in TCGA-CESC based on differences in different clinical indicators (Age, clinical_Stage, pathologic_T, pathologic_M, pathologic_N, histologic_Grade). On the other hand, the expression of CDA was significantly higher in age ≤ 50 group than age > 50 group, the expression of UPP1 was significantly higher I n pathologic M0 group than pathologic M1 group in CC (*p* < 0.05) (**Figure 7C**). The expression of SLC15A3 was significantly higher in grade 3 group than grade 2 group in OV (*p* < 0.01) (**Figure 7D**). The expressions of APOBEC3B and SLC15A3 were significantly higher and the expression of RHOB was significantly lower in grade 3 group than grade 2 group, and the expression of APOBEC3B was significantly higher in stage IV than stage I in UCEC (*p* < 0.05) (**Figure 7E**).

### 3.7 Function enrichment analyses of biomarkers in three common gynecologic cancers

**Figure 8:**
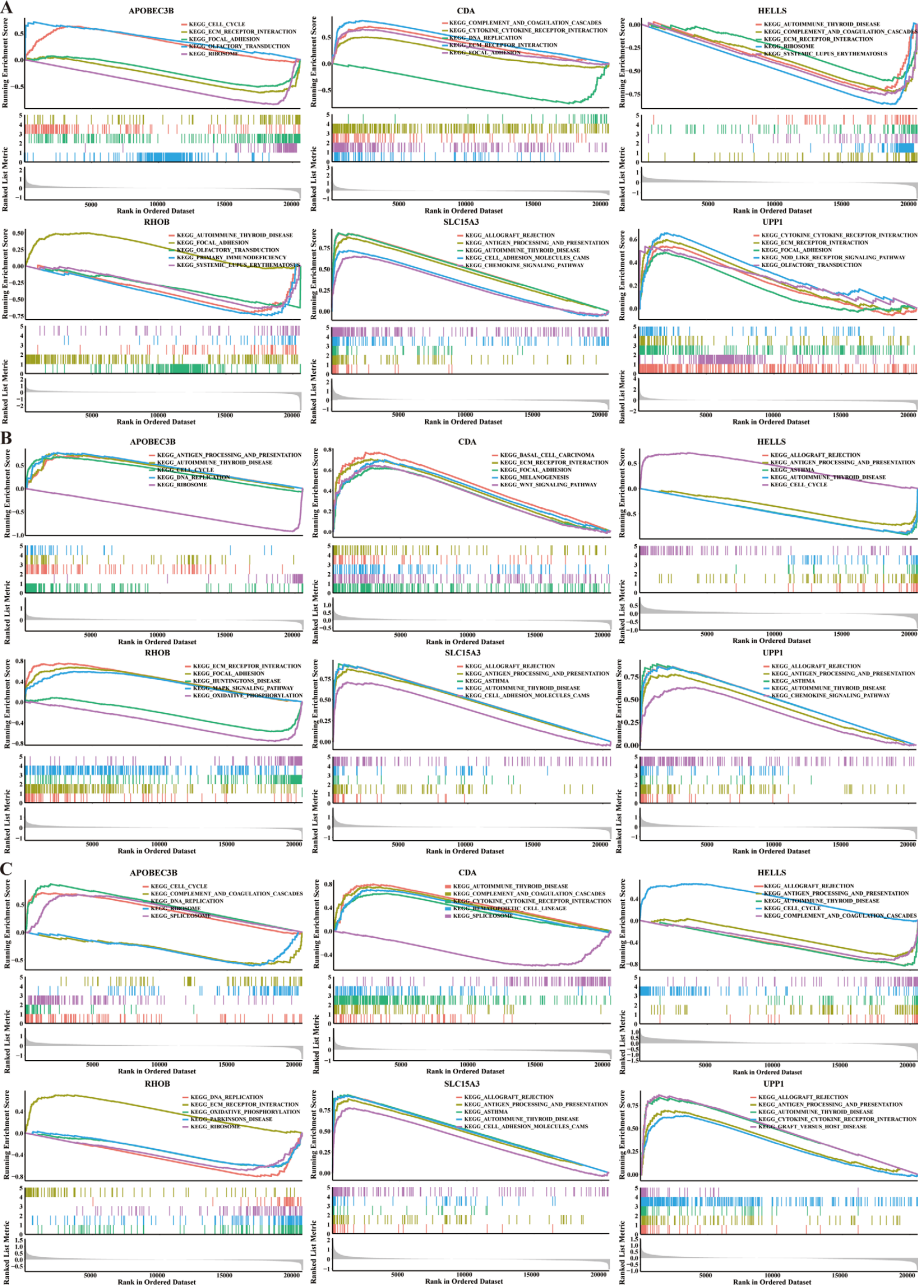
The GSEA of biomarkers in three common gynecologic cancers. **(A)** CC. **(B)** OV. **(C)** UCEC.

In CC, the ECM receptor interaction was significantly highly enriched in the high CDA, RHOB, and UPP1 expression groups and low APOBEC3B, HELLS, and SLC15A3 expression groups. Besides, all these biomarkers, except for RHOB, were associated with the complement and coagulation cascades, and all these biomarkers, except for SLC15A3, were associated with the focal adhesion (**Figure 8A**, **Supplementary Table 6**). In OV, all these biomarkers were associated with the antigen processing and presentation, cell adhesion, chemokine signaling pathway, and etc. KEGG pathways. Besides, all these biomarkers, except for APOBEC3B, were associated with the oxidative phosphorylation, leukocyte transendothelial migration, and JAK STAT signaling pathway, and all these biomarkers, except for HELLS, were associated with the focal adhesion (**Figure 8B**, **Supplementary Table 7**). In UCEC, the complement and coagulation cascades were significantly highly enriched in the high CDA, RHOB, and UPP1 expression groups and low APOBEC3B, HELLS, and SLC15A3 expression groups (**Figure 8C**, **Supplementary Table 8**).

On the other hand, we found that APOBEC3B was associated with the pathway of cell cycle in all these three common gynecologic cancers. CDA was associated with the pathways of ECM receptor interaction, focal adhesion, complement and coagulation cascades, cytokine receptor interaction, etc., in all these three common gynecologic cancers. HELLS was associated with the pathways of complement and coagulation cascades, cell cycle, cell adhesion, etc., in all these three common gynecologic cancers. RHOB was associated with the pathways of ECM receptor interaction, focal adhesion and etc. in all these three common gynecologic cancers. SLC15A3 was associated with the Toll like receptor, Nod like receptor, T cell receptor, B cell receptor, JAK STAT signaling pathways, FC gamma γ mediated phagocytosis and etc. in all these three common gynecologic cancers. UPP1 was associated with the pathways of Toll like receptor, Nod like receptor, JAK STAT, chemokine signaling pathways, ECM receptor interaction, focal adhesion, etc., in all these three common gynecologic cancers.

### 3.8 Mutation analyses of biomarkers in three common gynecologic cancers

**Figure 9:**
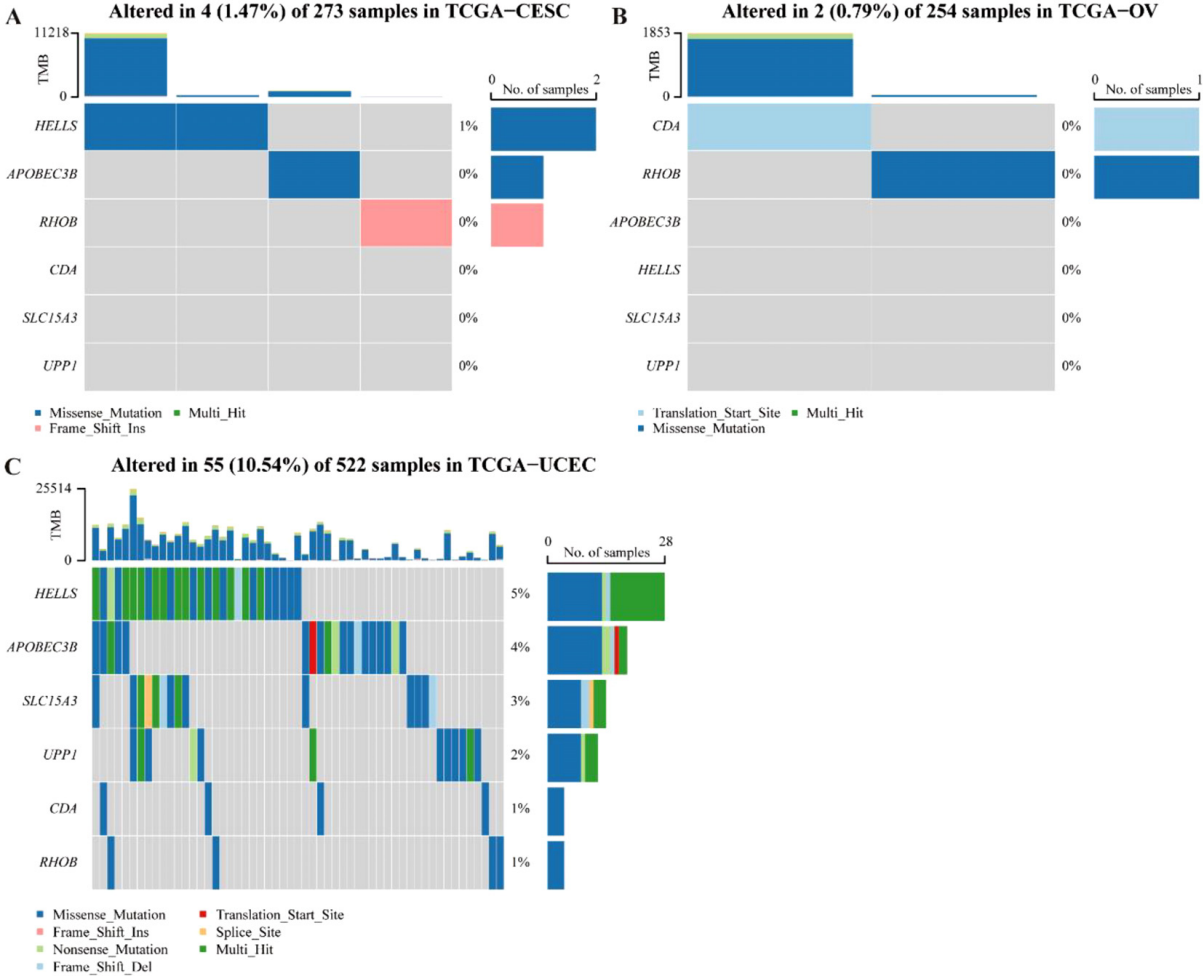
Mutation occurrence analyses of 6-CRRGs in three common gynecologic cancers. **(A)** CC. **(B)** OV. **(C)** UCEC.

In the end, the mutation situation of all these biomarkers in the three cancers was analyzed, and the results showed that missense mutation was the major mutation type in CC, OV, and UCEC. Besides, the mutation type of RHOB in CC was frame shift ins, the mutation type of CDA in OV was frameshift insertions (**Figures 9A-C**).

## 4 Discussion

Gynecological cancers have imposed a significant burden on women worldwide in terms of public health and the economy. Hermyt et al. (26) have reported that changes in miRNA activity may regulate the elevated expression of circadian rhythm genes NPAS2 and CSNK1D in endometrial cancer tissues. Additionally, they have observed that Clock and PER3 exhibit reduced expression in endometrial cancer tissues. Han et al. demonstrated that the overexpression of CRY1 and NANOG in cervical cancer is significantly associated with poor prognosis and resistance to chemoradiotherapy, indicating their potential as therapeutic targets for CC (27). Another CRRG, PER2, has been confirmed to be indirectly regulated by methylation and exhibits low expression in cervical cancer. Its overexpression inhibits the proliferation of drug-resistant cervical cancer cells through the PI3K/AKT pathway and promotes apoptosis, thereby improving the efficacy of cisplatin chemotherapy for CC (28). Yeh et al. discovered that ARNTL as a highly methylated target in ovarian cancer cells and demonstrated that its overexpression enhances the chemotherapy sensitivity of cisplatin (29). Therefore, it is imperative to explore the CRRGs associated with common gynecological cancers in order to identify potential therapeutic targets.

Bioinformatics is a field of study that integrates techniques from statistics, computer science, and biology. This interdisciplinary approach provides a comprehensive understanding of genetic data, potentially revealing new biological insights that may have been overlooked by single-discipline analyses. Bioinformatics has become an indispensable tool for modern biological research. Pathway analysis in bioinformatics enables the prediction of the effects of genetic variants on biological pathways, thereby providing greater contextual information about disease mechanisms (30). It can predict disease risk, identify drug targets and optimise drug dosing based on genetic information (31). Some studies have demonstrated that the use of bioinformatics analysis has the potential to identify abnormal *FAM64A* mRNA expression as a biomarker for oncogenesis, progression, invasiveness, and prognosis of gynecological malignancies (32). Similarly, abnormal BAG3 expression has the potential to serve as a marker for tumorigenesis, invasiveness, and prognosis, providing a new avenue for the treatment of cancers (33). Additionally, REG4 mRNA expression has been identified as a potential biomarker for gynecological cancers or a therapeutic target (34). Furthermore, it has been demonstrated that LASSO, in conjunction with bioinformatics analysis techniques, is a valuable approach for the identification of prognostic genes in gynecological cancers (35). This indicates that bioinformatics analysis methods are an effective means of predicting disease risk and identifying drug targets.

In this study, a multi-gene prognostic model based on the HCGA-CC cohort was constructed with good predictive performance, utilizing 6-CRRGs, including APOBEC3B, HELLS, SLC15A3, CDA, RHOB and UPP1. Furthermore, these biomarkers have been demonstrated to possess prognostic value in OV and UCEC. Previous studies have documented the overexpression of APOBEC3B in gynecological cancer (36–38). The overexpression of APOBEC3B has been demonstrated to induce TP53 gene mutation, which has been shown to significantly promote tumor cell proliferation, migration, chemotherapy resistance and recurrence through the p53 pathway (39, 40). HELLS was found to be highly expressed in a variety of cancer cells and serves as a poor prognostic biomarker. HELLS was overexpressed in cervical cancer and promoted the proliferation of cervical cancer by regulating the expression of nuclear factor erythroid 2-related factor 2 (Nrf2) (41). It has been demonstrated that HELLS plays a role in the promotion of homologous recombination of DNA double-strand breaks and contributes to the repair of heterochromatin regions during the G2 phase. The downregulation of HELLS has been demonstrated to inhibit tumor cell proliferation, colony formation, and induce G2/M cell cycle arrest, thereby representing a potential effective treatment for cervical cancer (42–44). UPP1 has been identified as a predictor of poor survival in cervical cancer, with a strong correlation to common carcinogenesis pathways and inflammation-related pathways. It plays a role in promoting tumor aggressiveness and predicting the effectiveness of immune checkpoint inhibitor therapy by modulating the immune microenvironment or mediating immune responses (45). To date, no relevant studies have been identified on other genes in cervical cancer. However, RHOB has been shown to play a role in the development of other cancers. RHOB maintains cell-cell adhesion in epithelial-derived cancer cells by regulating the levels and localization of E-cadherin. Downregulation of RHOB expression has been observed to enhance migration following a decrease in intercellular adhesion, thereby promoting tumor progression (46).

GSVA analysis of differentially expressed CRRGs revealed that the high-risk group was associated with the BMP signaling pathway, focal adhesion, and ECM receptor interaction pathways, among others. It has been demonstrated that the BMP signaling pathway is responsible for the occurrence, migration, and resistance to chemotherapy of ovarian cancer and endometrial cancer. This is achieved by regulating the stemness and epithelial-mesenchymal transition (EMT) of tumor cells. Conversely, the BMP signalling pathway has been shown to have a tumor-suppressing effect on CC (47). Gruber et al. demonstrated that the expression of β3-integrin was present in the majority of cervical cancer patients who underwent radiotherapy, and that the prognosis for patients with positive β3 integrin expression was significantly worse (48). The activated leukocyte cell adhesion molecule (ALCAM) is primarily involved in cell adhesion and signal transduction processes. Abnormal expression of ALCAM has been described in various tumors and is associated with cancer progression (49–51). Ihnen et al. have demonstrated that overexpression of ALCAM in cervical cancer tissue is associated with increased sensitivity to chemotherapy and radiotherapy (52). Zhang et al. found that Twist2 plays a role in the proliferation and invasion of renal cancer cells by regulating the expression of ITGA6 and CD44 in the ECM-receptor interaction pathway. In addition, abnormal activation of the ECM-receptor interaction signaling pathway may contribute to the development of breast cancer (53). Zhang et al. have shown that Twist2 is involved in the progression of renal cancer by regulating ITGA6 and CD44 in the ECM-receptor interaction pathway (54).

It has been demonstrated that demographic information has a significant impact on the risk and prognosis of gynecological cancers. Alimena et al. have assessed differences in cervical cancer survival by age and race, and have shown that young Black women are likely to have a higher stage of disease and lower overall survival (55). Recent studies have indicated the existence of racial/ethnic disparities in HPV infection, which may also be a contributing factor to the observed differences in cervical cancer (56). Furthermore, marital status is indirectly associated with the risk of gynecological cancer, exerting an influence on lifestyle, sexuality and mental health. It has been demonstrated that a woman’s marital status is associated with mortality from invasive cervical cancer infection (57). In our study, we combined clinical data and found that the CDA gene was highly expressed in age ≤ 50 group, which may be associated with early tumour development or progression. These findings enhance our comprehension of the biological mechanisms underlying gynecological cancers and furnish crucial insights for investigating the role of CDA in disease prognosis and treatment. They also provide robust scientific evidence in support of personalized and precise prevention, screening and treatment strategies for gynecological cancers.

The role of immune cells in the tumor microenvironment is a key area of research, with a particular focus on their potential influence on tumor occurrence, progression, prognosis, and tolerance to anticancer treatment (58). It has been demonstrated that circadian rhythm disorders are associated with the inhibition of immune cell infiltration (59). In our study, ssGSEA analysis revealed a notable decline in the abundance of seven distinct immune cell types, including activated B cells, activated CD4 T cells, activated CD8 T cells, effector memory CD8 T cells, immature dendritic cells, immature B cells, and MDSCs, in the high-risk group. Conversely, there was a notable increase in the neutrophil population. Matsumoto et al. observed a significant correlation between intratumoral neutrophil density and shorter progression-free survival (PFS) in patients with cervical cancer (CC) who underwent definitive radiotherapy (60). Subsequent analysis revealed that the high-risk group exhibited a reduced number of immune checkpoints, heightened immune escape, and diminished sensitivity to immunotherapy in comparison to the low-risk group. In contrast with our findings, Wang et al. observed that circadian rhythm genes were dysregulated in glioma, and anti-tumour immunocytes and immunosuppressive cells were significantly enriched in the high-risk group, which were more sensitive to immunotherapy (61). As reported by Chi et al., the majority of immune checkpoints were significantly upregulated in the low-risk group, indicating that patients in this group may benefit from immune checkpoint blockade (ICB) therapy (18). This finding aligns with our own observations. The rhythmic genes identified in our study offer promising avenues for immune checkpoint therapy in patients with gynecological malignancies and may lead to improved prognoses for patients.

The objective of our study is to elucidate the functional role of biomarkers associated with circadian rhythms in common gynecological cancers through a bioinformatics approach. Prior research has indicated a correlation between the severity of endometrial cancer (EC) and nocturnal work and rhythm disturbances (62). This is attributed to the involvement of clock genes (CG), microRNAs (miRNAs) and long non-coding RNAs (lncRNAs) in the etiology of EC (63). The risk of EC was significantly elevated among women who work long rotating night shifts, particularly in the presence of obesity, which doubles the risk (64, 65). Overexpression of the circadian rhythm gene NPAS2 was linked to unfavorable prognosis and clinicopathological characteristics of UCEC (66). In conclusion, the existing literature indicates that common gynecological cancers are associated with CRRGs. Furthermore, a recent study integrated the clinicopathological characteristics of CESC patients with risk scores to construct a Nomogram. The model validated the hypothesis that the 6-ANRG profile and its associated Nomogram may serve as a key factor in the management of patients with CESC (67). Another study constructed a risk score model containing 11 necroptosis-related lncRNAs, which showed potential in predicting the prognosis and immune response of CESC patients (68). The AUC values of the aforementioned models exceeded 0.6, which corroborates the findings of the present study. Collectively, these results substantiate the reliability and validity of these models in clinical applications. In light of the above, the risk model of circadian rhythm-related biomarkers in common gynecological cancers constructed in the present study is therefore deemed to be reliable.

The findings of our study contribute to the understanding of the mechanisms underlying the role of CRRGs in the development of common gynecological cancers. It should be noted, however, that the present study is not without limitations. Firstly, our study examined the expression of circadian rhythm-related genes at the transcriptome level, which may limit the generalizability of the findings in exploring interactions at the global transcript level. Secondly, this study employed a bioinformatics approach to investigate the functional role of biomarkers associated with circadian rhythms in common gynecological cancers. While the effectiveness of this analysis method has been demonstrated in numerous studies, the lack of validation experiments represents a limitation. As a result, the study will be repeated with a larger sample size, more effective methods will be employed, and the results will be further validated through additional experiments, including gene function experiments, immunoprecipitation experiments, protein expression analyses, and studies of transcriptional regulatory mechanisms. This will serve to enhance the depth and significance of the study. Furthermore, a significant number of the datasets employed in this study lack comprehensive clinical and demographic information, which precludes the assessment of the influence of socioeconomic factors on disease incidence and treatment outcomes. In the future, we will consider the impact of genetic variation across different racial and ethnic backgrounds, as well as the direct links between marital status and biological mechanisms, in order to assess the expression patterns of CRRGs in different groups. This could help to identify potential biological differences.

## 5 Conclusion

In conclusion, our findings indicate that the expression of 6-CRRGs, including APOBEC3B, HELLS, SLC15A3, CDA, RHOB and UPP1, is associated with the prognosis of patients with common gynecological cancers. This is due to their influence on ECM receptor interaction and focal adhesion pathways, among others. Our results may provide new insights into the prognostic mechanisms of common gynecological cancers and potential targets for immunotherapy.

## Data Availability

The original contributions presented in the study are included in the article/**Supplementary Material**. Further inquiries can be directed to the corresponding author/s.
